# Examining the association of changes in minimum wage with health across race/ethnicity and gender in the United States

**DOI:** 10.1186/s12889-019-7376-y

**Published:** 2019-08-08

**Authors:** Kimberly Danae Cauley Narain, Frederick J. Zimmerman

**Affiliations:** 1 UCLA Division of General Internal Medicine and Health Services Research, Department of Medicine, David Geffen School of Medicine and Center for Health Advancement, University of California, 1100 Glendon Ave., Suite 850, Los Angeles, CA 90024 USA; 2 Center for Health Advancement and Department of Health Policy and Management, Fielding School of Public Health, University of California, 10833 Le Conte Avenue, 31-236B CHS, Los Angeles, CA 90095 USA

**Keywords:** Minimum wage, Health disparities, Public policy, Race/ethnicity

## Abstract

**Background:**

The minimum wage creates both winners (through wage increases) and—potentially—losers (through job losses). Research on the health effects of minimum wage policies has been sparse, particularly across gender and among racial/ethnic minorities. We test the impact of minimum wage increases on health outcomes, health behaviors and access to healthcare across gender and race/ethnicity.

**Methods:**

Using 1993–2014 data from the Behavioral Risk Factor Surveillance System, variables for access to healthcare (insurance coverage, missed care due to cost), health behavior (exercise, fruit, vegetable and alcohol consumption) and health outcomes (self-reported fair/poor health, hypertension, poor physical health days, poor mental health days, unhealthy days) were regressed on the product of the ratio of the 1-year lagged minimum wage to the state median wage and the national median wage, using Linear Probability Models and Poisson Regression Models for dichotomous and count outcomes, respectively. Regressions (total population, gender-stratified, race/ethnicity stratified (white, black, Latino), gender/race/ethnicity stratified and total population with interaction terms for race/ethnicity/gender) controlled for state-level ecologic variables, individual-level demographics and fixed-effects (state and year). Results were adjusted for complex survey design and Bonferroni corrections were applied to *p*-values such that the level of statistical significance for a given outcome category was 0.05 divided by the number of outcomes in that category.

**Results:**

Minimum wage increases were positively associated with access to care among white men, black women and Latino women but negatively associated with access to care among white women and black men. With respect to dietary quality, minimum wage increases were associated with improvements, mixed results and negative impacts among white, Latino and black men, respectively. With respect to health outcomes, minimum wage increases were associated with positive, negative and mixed impacts among white women, white men and Latino men, respectively.

**Conclusions:**

While there is enthusiasm for minimum wage increases in the public health community, such increases may have to be paired with deliberate strategies to protect workers that might be vulnerable to economic dislocation. Such strategies may include more robust unemployment insurance or increased access to job training for displaced workers.

**Electronic supplementary material:**

The online version of this article (10.1186/s12889-019-7376-y) contains supplementary material, which is available to authorized users.

## Background

Federal law establishes a minimum wage for most types of workers throughout the country, and many states set a higher minimum wage [1]. Studies suggest that minimum wage impacts income in two different ways: increasing wages for employed low-wage workers and potentially reducing employment. However, it appears that the net effects are small [2–5]. Given the contribution of income to health, the effects of the minimum wage may well be important. Income underlies three major determinants of health: access to healthcare, environmental exposures and health behavior [6].

The relationship between minimum wage and health may be even more complex given that there may be differential income effects depending on gender, race and ethnicity. With respect to differential effects by gender, women constitute a larger proportion than men of workers either at or below minimum wage so women, particularly white women, could benefit more than men from minimum wage increases [7]. Racial and ethnic minorities may experience disparate employment loss associated with minimum wage increases as a consequence of workplace discrimination in layoffs and due to lower levels of human capital in the context of reduced employment demand [1, 8, 9]. Additionally there may be differential effects of the minimum wage across race/ethnicity and gender due to different patterns of distribution across job sectors [8]. Differential labor-market outcomes may result in differential health impacts across gender/racial/ethnic groups that are masked when looking at average effects.

The majority of studies of the health impacts of minimum wage explore average effects for all low income workers [10–12]. A few studies have explored the interplay of minimum wage and gender for health [10, 13, 14] and only two studies, to our knowledge, have examined the implications of gender, race/ethnicity and minimum wage for health [13, 14]. Andreyeva et al. (2018) explores this research question and does not find significant effects of minimum wage increases on racial/ethnic minorities (Latinos and Blacks); however, they do not disaggregate their results by racial/ethnic group and they do not explore if there are any interactions between race/ethnicity and gender [13]. Averett et al. (2017) explores this question in a cohort of teenagers and finds that minimum wage increases are associated with worse self-reported health among Latino men [14].

The objective of this study is to examine the effects of minimum wage on the access to care, health behaviors and health outcomes for different racial/ethnic/gender groups. This analysis provides empirical evidence that helps build out the conceptual framework for the effects of minimum wage laws on health by considering the effects on subpopulations in which this relationship has seldom been examined.

## Methods

### Data sample

To examine associations between minimum-wage policies access to care, health behaviors and health outcomes, among racial/ethnic minorities and across genders, this study analyzes a data from the Behavioral Risk Factor Surveillance System (BRFSS) and state-level data from other sources covering the 21-year period from 1993 to 2014. The BRFSS is conducted by state health departments, with technical and methodological assistance provided by the Centers for Disease Control and Prevention. This study sample includes individuals between the ages of 21 and 64. Consistent with the work of Horn et al. (2017), individuals who were homemakers, retired, self-employed, long-term unemployed, unable to work, and students were excluded from the sample. To focus on low-skill workers, the sample was limited to those individuals who reported educational attainment of high school (or General Educational Development test) or lower. A dataset was constructed consisting of pooled cross-sectional observations at the individual level, combined with state-level data on the prevailing minimum wage rate and several other policy and labor force characteristics that vary over time and across states. These ecological data were collected from the Bureau of Labor Statistics (BLS), Bureau of Economic Analysis and the University of Kentucky Center for Poverty Research [15–17]. This study was deemed exempt by the University of California, Los Angeles Institutional Review Board.

## Measures

Between 1993 and 2014 there were 313 minimum-wage changes (mostly increases) due to state and some federal legislation [10]. The state-specific median wage data used to construct the primary predictor are from the United States BLS [15]. We used the larger of the state or federal wage rate as the prevailing minimum wage and dates for minimum wage laws are based on the year of implementation.

The economic meaning of the minimum wage varies with local labor market conditions. A recent analysis studied the effects on the labor market of an increase in the federal minimum wage to $15/h, and found wide variations across states [18]. Some of this variation is due to differences in state minimum wages, but much is due to differences in state labor market conditions. In some states, many low-skill workers already earn very close to the proposed new federal minimum wage, whereas in other states very few do. For example, although the current state minimum wage is very similar in Connecticut ($10.10/h) and Minnesota ($9.86/h), the market wage for low-skill work is lower in Connecticut than in Minnesota, so that an increase to $15/h in both states would increase the wages of 26% of Connecticut workers and only 15% of Minnesotan workers. In Arkansas, where the state minimum wage is only slightly lower, at $9.25/h, 39% of the workforce would see an increase. Put differently, the state minimum wage is more economically binding in Arkansas than in Minnesota.

One way to capture these differences would be to divide the minimum wage by the local cost of living. But the cost of living, although correlated with average wages, is in fact not a factor in the low-skill labor market [19]. Therefore, economists have used a measure called the Kaitz index, which divides the minimum wage by the local average wage in the sector(s) most likely to be affected by the minimum wage [20, 21].

Our primary predictor is the Kaitz index with a slight modification to enhance interpretability: we multiply the ratio of the 1-year lagged minimum wage to the low-skill average wage) by the average national low-skill wage so that one unit can be generally understood as $1. The benchmark low-skill occupation is the state-specific median wage for first line supervisors/manager of sales workers (before 1999) and retail sales workers (after 1999). These occupations are chosen because they are typical low-skill positions that would be affected by the minimum wage and they have the most consistent definitions across time. In average-wage states, the regression coefficients can be interpreted as the effect of a $1 increase in the minimum wage. In high-wage states, the regression coefficients can be interpreted as the effect of an increase of more than $1, scaled to reflect the state’s higher-than-average wages. In low-wage states, the regression coefficients can be interpreted as the effect of an increase of somewhat less than $1. Additional file 1 reports these adjustment factors for all state-year combinations. Since most other studies exploring the relationship between minimum wage and health have used either the real or lagged minimum wage as their primary predictor we provide the results of models using the 1-year lagged minimum wage as a predictor as well; however, we will only be discussing the results derived from models including our main predictor, the adjusted wage ratio.

The two access to care variables, uninsurance (lack of health insurance) and missed care due to cost, were treated as indicator variables that were coded as “1” if the condition was present. Four health behavior outcomes were examined: 1 each pertaining to exercise and alcohol consumption and 2 pertaining to dietary intake (fruit and vegetables). An indicator for “no exercise in the last 30 days” was coded as “1” if the condition was present and the remaining health behavior variables for alcohol, fruit and vegetable consumption were count variables based on consumption during the last 30 days. We examine the amount of days alcohol was consumed in the last month because there is evidence that even moderate daily drinking may increase the risk of developing liver disease [22]. The health outcomes examined included 5 self-reported health outcomes: fair or poor health; diagnosis of hypertension; poor mental health days; poor physical health days; and unhealthy days. Self-reported fair or poor health and self-reported hypertension were indicator variables coded as “1” if the condition was present [23]. Self-reported poor mental health days and poor physical health days were treated as count variables based on the last 30 days. Following guidance from the Centers for Disease Control and Prevention, self-reported unhealthy days was a count variable constructed by combining the number of poor mental and physical health days [24, 25].

Included in the models are several time-varying, state-level covariates to control for possible confounding by local political environments. These variables were potentially associated both with the states’ decisions to modify a minimum-wage law and also with the health outcomes of interest. A variable for a state’s 1-year lagged Gross Domestic Product, using data obtained from the Bureau of Economic Analysis, reflects the economic environment [17]. Several variables characterize state-level welfare and assistance policies to capture the degree of state-specific generosity, including the percentage of the federal Earned Income Tax Credit that the state offers as a supplement, an indicator of whether this supplement is refundable and an indicator reflecting comprehensive Medicaid expansion to childless adults [26]. Two variables were included to capture state-level generosity in poverty policies: (1) the maximum monthly Temporary Assistance for Needy Families (TANF) benefit amount and (2) the maximum monthly food-stamp benefit amount, both for a family of 3. As a further control for possible confounding, the models also included state and year-level fixed effects in the models. The use of state-level fixed effects controls for any time-invariant state differences in political culture or economic environment that is unmeasured but that would otherwise confound the analysis. The inclusion of year-level fixed effects controls for any time trends that may be associated with changes to minimum wage policy. Lastly, several individual-level variables that may vary with time and have implications for self-reported health outcomes are included (age, race/ethnicity, gender, marital status, education and having minor children).

### Statistical analysis

We calculated the mean and standard error for our main predictor, adjusted minimum wage ratio, for each census region, over 5-year intervals and for the entire sample. We also calculated proportions and means for our dichotomous and count outcomes, respectively. The unit of analysis was the person-year for all analyses. Linear Regression Models and Poisson Regression Models (PRM) were estimated for dichotomous and count outcomes, respectively. Linear regression applied to dichotomous outcomes, also known as a Linear Probability Model (LPM), can be used when the sample size is large, since the distribution of the variables can be assumed to be normal under these circumstances [27]. Use of the LPM also allows direct interpretation of the coefficient of the primary predictor. We use a PRM to model count outcomes instead of an Ordinary Least Squared regression model because the normality assumption may not be applied to count data even in the context of large sample sizes if the number of counts is small. Additionally, use of the PRM will account for the non-linearity of count data [28]. All models were weighted to account for complex survey design and non-response by using the sampling weights provided by the BRFSS. Additionally, robust standard errors clustered at the state level were used. We employed these adjustments through the SVY command with the linearized option in Stata version 13 (StataCorp LP, College Station, TX). All monetary variables are adjusted for inflation. We initially ran models for each outcome inclusive of the entire sample and then we ran models stratified by gender. Next we ran models stratified by race/ethnicity (white, black and Latino) and models stratified by both race/ethnicity and gender. Lastly, we ran analyses for the total population including interaction terms for race/ethnicity/gender and the adjusted wage ratio. In addition to these main analyses, we ran models stratified by race/ethnicity/gender and high vs. low wage state and analyses stratified race/ethnicity/gender and marital status. The results of these analyses can be found in appendices 3–6 and 7–10, respectively. In order to adjust the level of statistical significance for multiple comparisons, we apply the Bonferroni correction such that the level of statistical significance for a given outcome category is 0.05 divided by the number of outcomes in that category (access to care (2): *p*-value≤0.025, health behavior (4): *p*-value≤0.0125 and health outcomes (5): *p*-value≤ .010) [29]. The results of the LPMs and PRMs are presented as marginal effects and rate ratios, respectively.

## Results

A table showing the adjusted wage ratio by time and census region can be found in Appendix 2. The full-sample mean of the adjusted wage ratio variable is 6.11 and the interquartile range is 3.18–9.37. The descriptive statistics for the covariates and outcomes are found in Table 1.

**Table 1:** Descriptive Statistics

	*Mean (SD) or %*
*Access to Care*	
Uninsurance	26.2
Missed care due to cost	20.2
*Health Behaviors*	
No exercise in the last 30 days	32.9
^1^Days ETOH consumed in the last 30 days	5.2 (6.6)
Times fruit consumed in the last 30 days	23.1 (26.7)
Vegetable servings consumption in the last 30 days	26.3 (23.0)
*Health Outcomes*	
Self-reported hypertension	25.4
Self-reported fair or poor health	16.9
Poor mental health days in the last 30 days	3.7 (6.9)
Poor physical health days in the last 30 days	2.8 (6.0)
Unhealthy days in the last 30 days	5.9 (8.4)
*State-level variables*	
State Earned Income Tax Rate	.04 (.07)
^2^State EITC is Refundable	28.1
Maximum Temporary Needy Assistance Benefit for 3	390 (140)
Maximum Food Stamp Benefit for 3	423 (31)
1-year lagged Gross Domestic Product %	4.1 (2.4)
Medicaid Expansion to Childless Adults	9.2
*Individual-level variables*	
High School Diploma/GED	73.7
Age	40.5 (10.2)
Black	12.3
Latino	20.8
Female	42.5
Married	56.9
Minor Children	51.9

1. ETOH = Alcohol. 2. EITC = Earned Income Tax Credit. All estimates are weighted to reflect sample design

### Results for all races (Total Population & across Gender)

The results capturing the impact of minimum wage increases, on the access to care, health behaviors and health outcomes for all races (total population and stratified by gender) can be found in Table 2. Among the total population, a $1 increase in the minimum wage reduced the probability of reporting no insurance and care cost by 2 (β = −.02; 95% CI = -.03 to −.02), and 1 percentage points (β = −.01; 95% CI = -.01 to −.00), respectively, in average wage states, controlling for other factors. With respect to health behaviors, a $1 increase in minimum wage increased the expected number of times fruit was consumed and servings of vegetables were consumed by 3% (β = 1.03; 95% CI =1.00 to 1.06), and 5% (β = 1.05; 95% CI =1.03 to 1.07), respectively, in average wage states.

**Table 2:** The Relationship of Minimum Wage, Access to Care, Health Behaviors and Health (All Races)

Outcome	Total		Men		Women	
	Wage Ratio	Minimum Wage	Wage Ratio	Minimum Wage	Wage Ratio	Minimum Wage
Access to Care						
No Health Insurance^1^	**-.02** (−.03,-.02) *N* = 869,908	**-.03** (−.04,-.02) *N* = 869,908	**-.03** (−.04,-.02) *N* = 399,604	**-.04** (−.05, −.03) *N* = 399,604	**−.01** (−.02,-.01) *N* = 470,304	**−.02** (−.03,−.01) *N* = 470,304
Missed care due to cost^1^	**−.01** (−.01,-.00) *N* = 822,864	**−.01** (−.02,-.01) *N* = 822,864	**-.01** (−.02, −.00) *N* = 378,071	**−.02** (−.03, −.01) *N* = 378,071	−.01 (−.02,.00) *N* = 444,793	**−.01** (−.02, −.00) *N* = 444,793
Health Behavior						
No exercise^1^	−.00 (−.01,.01) *N* = 781,929	.01 (−.00,.02) *N* = 781,929	.00 (−.01, .01) *N* = 358,495	.01 (−.01, .02) *N* = 358,495	−.01 (−.02,.00) *N* = 423,434	.00 (−.01,.02) *N* = 423,434
Fruit Consumption^2^	**1.03** (1.00,1.06) *N* = 446,162	1.01 (.98,1.04) N = 446,162	1.04 (.99,1.08) *N* = 203,157	1.02 (.97,1.06) *N* = 203,157	1.02 (.99,1.05) *N* = 243,005	1.00 (.96,1.04) *N* = 243,005
Vegetable Consumption^2^	**1.05** (1.03,1.07) *N* = 443,602	1.01 (.99,1.03) *N* = 443,602	**1.05** (1.02,1.08) *N* = 201,663	1.01 (.97,1.04) *N* = 201,663	**1.05** (1.02,1.08) *N* = 241,939	1.01 (.99,1.04) *N* = 241,939
Alcohol Consumption^2^	1.01 (.98,1.03) *N* = 554,781	1.00 (.96,1.03) *N* = 554,781	1.01 (.97,1.04) *N* = 273,817	.99 (.95–1.03) *N* = 273,817	1.01 (.97,1.05) *N* = 280,964	1.00 (.96,1.05) *N* = 280,964
Health Outcomes						
Self-reported poor health^1^	.00 (−.00,.01) *N* = 869,457	−.00 (−.01,.01) *N* = 869,457	.01 (−.00,.02) *N* = 399,603	.00 (−.01,.01) *N* = 399,603	−.00 (−.01,.00) *N* = 469,854	−.01 (−.02,.00) *N* = 469,854
Self-reported HTN^1^	.00 (−.00,.01) *N* = 474,408	.00 (−.01,.02) *N* = 474,408	.01 (−.01,.02) *N* = 217,249	.01 (−.01,.02) *N* = 217,249	.00 (−.01,.01) *N* = 257,159	.00 (−.01,.02) *N* = 257,159
Unhealthy Days^2^	1.02 (.99,1.05) *N* = 858,345	1.00 (.97,1.04) *N* = 858,345	1.03 (.99,1.07) *N* = 394,779	1.01 (.96,1.07) *N* = 394,779	1.01 (.98,1.04) *N* = 463,566	.99 (.96,1.03) *N* = 463,566
Poor Mental Health Days^2^	1.03 (.99,1.07) *N* = 848,897	1.01 (.97,1.05) *N* = 848,897	1.05 (.99,1.11) *N* = 390,704	1.03 (.96,1.10) *N* = 390,704	1.01 (.97,1.05) *N* = 458,193	.99 (.94,1.04) *N* = 458,193
Poor Physical Health Days^2^	1.02 (.98,1.06) *N* = 849,354	1.00 (.96,1.05) *N* = 849,354	1.02 (.96,1.09) *N* = 391,031	1.01 (.94,1.09) *N* = 391,031	1.01 (.97,1.06) *N* = 458,323	.99 (.94,1.05) *N* = 458,323

The data source is BRFSS (1993–2014 panels). Linear Probability Models and Poisson Regression Models are used to examine dichotomous and count outcomes, respectively. All models control for state earned income tax credit rate, refundability of state earned income tax credit, Maximum food stamp allotment for a family of 3 maximum TANF allotment for a family of 3, 1-year lagged GDP, comprehensive Medicaid expansion, age, race/ethnicity, marital status, education and having minor children, year as well as state fixed-effects. All models are weighted for complex survey design and non-response. Total population models also control for gender. Standard errors are robust and clustered at the state level. Results of LPMs and PRMs are presented as percentage point differences in the probability of an outcome and Rate Ratios, respectively. All monetary values are inflation-adjusted. Boldface indicates statistical significance. Significance levels: *(access to care: *p*-value-.025, health behaviors: *p*-value-.0125, and health outcomes: *p*-value .010) (Bonferroni-corrected 95% confidence Intervals in parenthesis). Notes: 1. Marginal effect 2. Rate Ratio; Bold indicates statistical significance

Among men, minimum wage increases were linked with decreased reports of no health insurance (β = −.03; 95% CI = -.04 to −.02), and missed care due to cost (β = −.01; 95% CI = −.02 to −.00) as well as increased vegetable consumption (β = 1.05; 95% CI =1.02 to 1.08).

Among women, minimum wage increases were associated with reduced reports of no insurance (β = −.01; 95% CI = -.02 to −.01) and increased reports of vegetable consumption (β = 1.05; 95% CI =1.02 to 1.08).

### Results for whites (Total Population & across Gender)

The results of stratified models for whites are presented in Table 3 and the results of the models using interaction terms are found in Table 4. In stratified models, minimum wage increases were associated with reduced reporting of missed care due to cost (β = −.01; 95% CI = -.01 to −.00) and increased vegetable consumption (β = 1.03; 95% CI =1.00 to 1.05), among the total white population. Among white men, there is a positive association between minimum wage increases and reporting no exercise (β = .01; 95% CI = .00 to .02), vegetable consumption (β = 1.03; 95% CI =1.00 to 1.06) and self-reported fair/poor health (β = .01; 95% CI = .00 to .02). In models with interaction terms, the impacts of minimum wage increases are associated with reduced reports of no insurance (β = −.02; 95% CI = −.03 to −.02) and missed care due to cost, (β = −.01; 95% CI = -.02 to −.01) and positively associated with vegetable consumption (β = 1.05; 95% CI =1.02 to 1.07) and poor mental health days (β = 1.04; 95% CI =1.00 to 1.08). The minimum wage effects on no exercise and self-reported fair/poor health are no longer significant.

**Table 3:** The Relationship of Minimum Wage, Access to Care, Health Behaviors and Health (Whites)

Outcome	Total		Men		Women	
	Wage Ratio	Minimum Wage	Wage Ratio	Minimum Wage	Wage Ratio	Minimum Wage
Access to Care						
No Health Insurance^1^	−.00 (−.01,.00) *N* = 623,932	**−.01** −.02,−.00) *N* = 623,932	-.00 (−.01,.01) *N* = 289,254	−.01 (−.02,.00) *N* = 289,254	−.00 (−.01,.00) *N* = 334,678	−.01 (−.02, .00) *N* = 334,678
Missed care due to cost^1^	**−.01** (−.01,-.00) *N* = 589,983	**−.01** (−.02,-.00) *N* = 589,983	−.01 (−.01,.00) *N* = 273,535	**−.01** (−.02, −.00) *N* = 273,535	−.01 (−.02, .00) *N* = 316,448	**−.01** (−.02,-.00) *N* = 316,448
Health Behavior						
No exercise^1^	.00 (−.00,.01) *N* = 557,307	.01 (.00,.02) *N* = 557,307	**.01** (.00,.02) *N* = 257,669	.02 (.00,.03) *N* = 257,669	−.01 (−.02,.00) *N* = 299,638	.00 (−.01,.02) *N* = 299,638
Fruit Consumption^2^	**1.02** (1.00,1.05) *N* = 322,351	1.00 (.97,1.04) *N* = 322,351	1.04 (.99,1.08) *N* = 148,088	1.01 (.96,1.06) *N* = 148,088	1.01 (.98,1.04) *N* = 174,263	1.00 (.96,1.04) *N* = 174,263
Vegetable Consumption^2^	**1.03** (1.01,1.05) *N* = 321,288	1.00 (.98,1.02) *N* = 321,288	**1.03** (1.00,1.06) *N* = 147,452	.99 (.96,1.03) *N* = 147,452	**1.03** (1.00,1.05) *N* = 173,836	1.00 (.98,1.03) *N* = 173,836
Alcohol Consumption^2^	1.01 (.98,1.03) *N* = 404,016	1.00 (.97,1.03) *N* = 404,016	1.01 (.97, 1.04) *N* = 199,207	1.00 (.95,1.04) *N* = 199,207	1.01 (.97,1.05) *N* = 204,809	1.01 (.96,1.06) *N* = 204,809
Health Outcomes						
Self-reported poor health^1^	.00 (−.00,.01) *N* = 623,682	−.00 (−.01,.01) *N* = 623,682	**.01** (.00,.02) *N* = 289,243	.01 (−.00,.02) *N* = 289,243	**−.01** (−.01,-.00) *N* = 334,439	**−.01** (−.02,−.00) *N* = 334,439
Self-reported HTN^1^	.00 (−.01,.01) *N* = 343,549	−.00 (−.01,.01) *N* = 343,549	.00 (−.01,.02) *N* = 158,946	−.00 (−.02,.01) *N* = 158,946	−.00 (−.01,.01) *N* = 184,603	−.00 (−.02,.01) *N* = 184,603
Unhealthy Days^2^	1.01 (.98,1.03) *N* = 616,008	.99 (.96,1.03) *N* = 616,008	1.02 (.97,1.06) *N* = 285,904	1.00 (.94,1.06) *N* = 285,904	1.00 (.96,1.03) *N* = 330,104	.98 (.94,1.02) *N* = 330,104
Poor Mental Health Days^2^	1.00 (.96, 1.03) *N* = 609,531	.97 (.93,1.02) *N* = 609,531	1.00 (.94,1.06) *N* = 283,124	.98 (.91,1.05) *N* = 283,124	.99 (.95,1.03) *N* = 326,407	.97 (.92,1.02) *N* = 326,407
Poor Physical Health Days^2^	1.02 (.98,1.07) *N* = 610,595	1.01 (.96,1.06) *N* = 610,595	1.04 (.97,1.11) *N* = 283,629	1.02 (.94,1.11) *N* = 283,629	1.00 (.95,1.06) *N* = 326,966	.99 (.93,1.06) *N* = 326,966

The data source is BRFSS (1993–2014 panels). Linear Probability Models and Poisson Regression Models are used to examine dichotomous and count outcomes, respectively. All models control for state earned income tax credit rate, refundability of state earned income tax credit, Maximum food stamp allotment for a family of 3 maximum TANF allotment for a family of 3, 1-year lagged GDP, comprehensive Medicaid expansion, age, marital status, education and having minor children, year as well as state fixed-effects. All models are weighted for complex survey design and non-response. Total population models also control for gender. Standard errors are robust and clustered at the state level. Results of LPMs and PRMs are presented as percentage point differences in the probability of an outcome and Rate Ratios, respectively. All monetary values are inflation-adjusted. Boldface indicates statistical significance. Significance levels: *(access to care: *p*-value-.025, health behaviors: *p*-value−.0125, and health outcomes: *p*-value .010) (Bonferroni-corrected 95% confidence intervals in parenthesis). Notes: 1. Marginal effect 2. Rate Ratio; Bold indicates statistical significance

**Table 4:** The Relationship of Minimum Wage to Access to Care, Health Behaviors and Health Outcomes

	Access to care		Health Behaviors				Health Outcomes				
	% Point Difference in No Health Insurance *N* = 869,908	% Point Difference in Missed Care due to Cost *N* = 822,864	% Point Difference in No Exercise *N* = 781,929	Fruit Consumption (rate ratio) *N* = 446,162	Vegetable consumption (rate ratio) *N* = 443,602	Alcohol Consumption (rate ratio) *N* = 554,781	% Point Difference in Self-Reported Fair/Poor Health N = 869,457	% Point Difference In Self-Reported HTN *N* = 474,408	Unhealthy Days (rate ratio) *N* = 858,345	Poor Mental Health Days (rate ratio) *N* = 848,897	Poor Physical Health Days (rate ratio) *N* = 849,354
Adjusted Wage Ratio (MW)	**-.02**	**-.01**	.00	1.03	**1.05**	1.01	.00	.01	1.03	**1.04**	1.03
Female	**−.04**	.01	**.03**	1.15	**1.07**	**.60**	−.00	−.00	**1.61**	**1.75**	**1.49**
Black	**−.10**	**−.04**	.04	1.09	**1.55**	1.16	.01	.06	.85	.84	.92
Latino	**.25**	−.04	**.27**	**1.70**	**.55**	**.71**	**.19**	−.04	**.55**	**.58**	**.59**
Female*Black	**.09**	.01	**.09**	.92	.99	**.71**	−.02	.03	.96	.92	**.89**
Female *Latino	−.06	**.20**	−.00	.74	1.10	.96	−.03	.08	1.17	1.06	**1.15**
MW*Female	.00	**.01**	.00	1.01	1.01	.99	.00	**−.01**	**.98**	**.98**	**.97**
MW*Black	**.02**	**.01**	−.00	1.00	**.92**	**.96**	.00	−.00	1.02	1.02	1.02
MW*Latino	**−.01**	**.01**	**−.03**	**.96**	**1.05**	.99	−.01	−.00	**1.06**	1.03	**1.06**
MW*Female* Black	**−.02**	−.00	−.00	1.01	.99	1.04	.01	.01	.99	.99	1.00
MW*Female* Latino	.00	**−.03**	.00	1.04	1.00	.97	.01	−.00	.98	.99	.99

The data source is BRFSS (1993–2014 panels). Linear Probability Models and Poisson Regression Models are used to examine dichotomous and count outcomes, respectively. All models control for state earned income tax credit rate, refundability of state earned income tax credit, Maximum food stamp allotment for a family of 3 maximum TANF allotment for a family of 3, 1-year lagged GDP, comprehensive Medicaid expansion, age, marital status, education and having minor children, and year as well as state fixed-effects. All models are weighted for complex survey design and non-response. Standard errors are robust and clustered at the state level. Results of LPMs and PRMs are presented as percentage point differences in the probability of an outcome and Rate Ratios, respectively. All monetary values are inflation-adjusted. Boldface indicates statistical significance. Significance levels: *(access to care: *p*-value-.025, health behaviors: *p*-value-.0125, and health outcomes: *p*-value .010)

In stratified models including white women, minimum wage increases are positively associated with vegetable consumption (β = 1.03; 95% CI =1.00 to 1.05) and decreased reporting of fair/poor health (β = −.01; 95% CI = -.01 to −.00). In the models with interactions terms, minimum wage increases are positively linked to missed care due to cost (β = .01; 95% CI = .01 to .01), but negatively linked with reports of hypertension (β = −.01; 95% CI = -.01 to- .00), poor mental health days (β = .98; 95% CI = .96 to .99), poor physical health days (β = .97; 95% CI = .95 to .99) and unhealthy days (β = .98; 95% CI = .96 to .99). The relationships between minimum wage, self-reported fair/poor health and vegetable consumption are no longer significant.

### Results for blacks (Total Population & across Gender)

The results of the stratified models, conducted in the black population (total, men or women) (Table 5), did not reveal any statistically significant relationship between minimum wage increases and any of the access to care, health behavior or health outcomes; however, in models using interaction terms (Table 4), minimum wage increases were found to be positively associated with reports of no health insurance (β = .02; 95% CI = .02 to .03) and missed care due to cost (β = .01; 95% CI = .00 to .02) but negatively associated with vegetable (β = .92; 95% CI = .89 to .94) and alcohol consumption (β = .96; 95% CI = .93 to .99) among black men. Among black women, minimum wage increases were found to be negatively associated with reports of no health insurance (β = −.02; 95% CI = −.03 to −.01).

**Table 5:** The Relationship of Minimum Wage, Access to Care, Health Behaviors and Health (Blacks)

Outcome	Total		Men		Women	
	Wage Ratio	Minimum Wage	Wage Ratio	Minimum Wage	Wage Ratio	Minimum Wage
Access to Care						
No Health Insurance^1^	.01 (−.01,.03) *N* = 95,091	-.00 (−.03,.02) *N* = 95,091	.02 (−.01,.05) *N* = 34,746	.01 (−.03,.05) *N* = 34,746	-.01 (−.03,.02) *N* = 60,345	−.02 (−.05,.01) *N* = 60,345
Missed care due to cost^1^	.01 (−.01,.02) *N* = 90,096	.00 (−.02,.03) *N* = 90,096	.01 (−.02,.04) *N* = 32,911	.00 (−.04,.04) *N* = 32,911	.00 (−.02,.03) *N* = 57,185	.00 (−.03,.04) *N* = 57,185
Health Behavior						
No exercise^1^	−.01 (−.03, .02) *N* = 85,243	.01 (−.02,.05) N = 85,243	−.01 (−.05,.03) *N* = 31,022	.01 (−.04,.05) N = 31,022	−.00 (−.03,.03) *N* = 54,221	.02 (−.02,.07) N = 54,221
Fruit Consumption^2^	1.00 (.92,1.09) *N* = 48,174	.98 (.88,1.09) *N* = 48,174	.92 (.81,1.05) *N* = 17,522	.88 (.75,1.03) *N* = 17,522	1.08 (.97,1.20) *N* = 30,652	1.08 (.93,1.24) *N* = 30,652
Vegetable Consumption^2^	1.01 (.95,1.08) *N* = 48,022	.95 (.88,1.03) *N* = 48,022	1.01 (.90,1.12) *N* = 17,459	.92 (.81,1.06) *N* = 17,459	1.02 (.94,1.11) *N* = 30,563	.98 (.88,1.08) *N* = 30,563
Alcohol Consumption^2^	.98 (.89,1.07) *N* = 55,024	.99 (.88,1.12) *N* = 55,024	.94 (.83,1.06) *N* = 22,138	.98 (.84,1.15) *N* = 22,138	1.06 (.93,1.20) *N* = 32,886	.99 (.84,1.17) *N* = 32,886
Health Outcomes						
Self-reported poor health^1^	.02 (−.00, .03) *N* = 94,980	.01 (−.01,.04) *N* = 94,980	.02 (−.01,.05) *N* = 34,735	.02 (−.02,.06) *N* = 34,735	.01 (−.02,.03) *N* = 60,245	.01 (−.02, .04) *N* = 60,245
Self-reported HTN^1^	−.00 (−.03,.03) *N* = 52,093	−.00 (−.04,.04) *N* = 52,093	.03 (−.02,.08) *N* = 19,074	.03 (−.03,.09) *N* = 19,074	−.03 (−.07,.00) *N* = 33,019	−.03 (−.07,.02) *N* = 33,019
Unhealthy Days^2^	1.01 (.93,1.10) *N* = 93,097	1.01 (.90,1.12) *N* = 93,097	1.02 (.88,1.18) *N* = 34,046	1.01 (.84,1.22) *N* = 34,046	1.00 (.91,1.10) *N* = 59,051	1.00 (.88,1.13) *N* = 59,051
Poor Mental Health Days^2^	1.04 (.93,1.16) *N* = 92,108	1.02 (.89,1.18) *N* = 92,108	1.11 (.91,1.35) *N* = 33,667	1.11 (.86,1.44) *N* = 33,667	.99 (.88,1.11) *N* = 58,441	.96 (.82,1.12) *N* = 58,441
Poor Physical Health Days^2^	1.01 (.89,1.14) *N* = 91,682	1.04 (.89,1.22) *N* = 91,682	.97 (.80,1.18) *N* = 33,570	.99 (.76,1.29) *N* = 33,570	1.03 (.89,1.19) *N* = 58,112	1.08 (.89,1.30) *N* = 58,112

The data source is BRFSS (1993–2014 panels). Linear Probability Models and Poisson Regression Models are used to examine dichotomous and count outcomes, respectively. All models control for state earned income tax credit rate, refundability of state earned income tax credit, Maximum food stamp allotment for a family of 3 maximum TANF allotment for a family of 3, 1-year lagged GDP, comprehensive Medicaid expansion, age, marital status, education and having minor children, year as well as state fixed-effects. All models are weighted for complex survey design and non-response. Total population models also control for gender. Standard errors are robust and clustered at the state level. Results of LPMs and PRMs are presented as percentage point differences in the probability of an outcome and Rate Ratios, respectively. All monetary values are inflation-adjusted. Boldface indicates statistical significance. Significance levels: *(access to care: *p*-value−.025, health behaviors: *p*-value−.0125, and health outcomes: *p*-value .010) (Bonferroni-corrected 95% confidence Intervals in parenthesis). Notes: 1. Marginal effect 2. Rate Ratio; Bold indicates statistical significance

### Results for Latinos (Total Population & across Gender)

In stratified models of the total Latino population (Table 6), minimum wage increases were positively associated with fruit intake (β = 1.10; 95% CI =1.01 to 1.21), poor physical health days (β = 1.15; 95% CI =1.02 to 1.30) and unhealthy days (β = 1.14; 95% CI =1.00 to 1.30). Among Latino men, minimum wage increases were associated with reduced reports of no health insurance (β = −.03; 95% CI = -.06 to −.00) as well as increased fruit consumption (β = 1.10; 95% CI =1.01 to 1.21) and reports of poor physical health days (β = 1.19; 95% CI =1.00 to 1.41). In models using interaction terms, minimum wage increases were negatively associated with reports of no health insurance (β = −.01; 95% CI = -.02 to −.00), no exercise (β = −.03; 95% CI = −.04 to −.02), fruit consumption (β = .96; 95% CI = .92 to .99) and self-reported fair/poor health (β = −.01; 95% CI = -.02 to −.00) and positively associated with vegetable consumption (β = 1.05; 95% CI =1.01 to 1.08), missed care due to cost, (β = .01; 95% CI = .02 to .00), poor physical health days (β = 1.06; 95% CI =1.00 to 1.13) and unhealthy days (β = 1.06; 95% CI =1.01 to 1.10). No statistically significant relationship were observed for any outcomes among Latino women in stratified models; however, models using interaction terms showed reduced reports of missed care due to cost among Latino women associated with minimum wage increases (β = −.03; 95% CI = -.04 to −.01) .

**Table 6:** The relationship of minimum wage, access to care, health behaviors and health (Latinos)

Outcome	Total		Men		Women	
	Wage Ratio	Minimum Wage	Wage Ratio	Minimum Wage	Wage Ratio	Minimum Wage
Access to Care						
No Health Insurance^1^	-.02 (−.04, .00) *N* = 81,286	-.01 (−.04, .01) *N* = 81,286	**−.03** (−.06,-.00) *N* = 40,809	−.03 (−.06,.01) *N* = 40,809	−.00 (−.04, .03) *N* = 40,477	.01 (−.02,.05) *N* = 40,477
Missed care due to cost^1^	−.00 (−.02,.02) *N* = 76,729	−.01 (−.03,.02) *N* = 76,729	−.01 (−.04,.02) *N* = 38,546	−.02 (−.05,.01) *N* = 38,546	.02 (−.01,.05) *N* = 38,183	.02 (−.01,.06) *N* = 38,183
Health Behavior						
No exercise^1^	.00 (−.02,.03) *N* = 79,855	−.01 (−.04,.02) *N* = 79,855	.00 (−.03,.04) *N* = 40,049	−.01 (−.05,.03) *N* = 40,049	−.00 (−.04,.03) *N* = 39,806	−.01 (−.05,.03) *N* = 39,806
Fruit Consumption^2^	**1.10** (1.01,1.21) *N* = 38,035	1.10 (.99,1.23) *N* = 38,035	**1.16** (1.03,1.31) *N* = 18,888	**1.17** (1.01,1.36) *N* = 18,888	1.03 (.90,1.17) *N* = 19,147	1.00 (.86,1.16) *N* = 19,147
Vegetable Consumption^2^	1.04 (.96,1.13) *N* = 37,186	.98 (.89,1.07) *N* = 37,186	1.04 (.93,1.16) *N* = 18,387	1.00 (.88,1.14) *N* = 18,387	1.03 (.93,1.15) *N* = 18,799	.94 (.83,1.06) *N* = 18,799
Alcohol Consumption^2^	.98 (.88,1.09) *N* = 56,398	.92 (.81,1.04) *N* = 56,398	.99 (.87,1.11) *N* = 30,959	.91 (.79,1.05) *N* = 30,959	.92 (.77,1.08) *N* = 25,439	.94 (.75,1.18) *N* = 25,439
Health Outcomes						
Self-reported poor health^1^	−.00 (−.02,.02) *N* = 81,113	−.01 (−.03,.02) *N* = 81,113	−.00 (−.03,.03) *N* = 40,734	−.01 (−.05,.03) *N* = 40,734	.01 (−.03,.04) *N* = 40,379	.00 (−.03,.04) *N* = 40,379
Self-reported HTN^1^	.00 (−.03,.03) *N* = 42,576	.00 (−.03,.04) *N* = 42,576	−.01 (−.05,.02) *N* = 21,225	.00 (−.04,.04) *N* = 21,225	.03 (−.01,.07) *N* = 21,351	.01 (−.04,.05) *N* = 21,351
Unhealthy Days^2^	**1.11** (1.02,1.21) *N* = 80,129	1.03 (.92,1.14) *N* = 80,129	1.14 (.99,1.30) *N* = 40,212	1.06 (.90,1.24) *N* = 40,212	1.08 (.96,1.21) *N* = 39,917	1.00 (.87,1.14) *N* = 39,917
Poor Mental Health Days^2^	1.10 (.97,1.24) *N* = 79,127	.99 (.85,1.15) *N* = 79,127	1.14 (.94,1.37) *N* = 39,742	1.01 (.80,1.27) *N* = 39,742	1.06 (.90,1.23) *N* = 39,385	.97 (.81,1.16) *N* = 39,385
Poor Physical Health Days^2^	**1.15** (1.03,1.30) *N* = 78,963	1.10 (.96,1.27) *N* = 78,963	**1.19** (1.00,1.41) *N* = 39,659	1.15 (.94,1.41) *N* = 39,659	1.11 (.96,1.29) *N* = 39,304	1.05 (.88,1.25) *N* = 39,304

The data source is BRFSS (1993–2014 panels). Linear Probability Models and Poisson Regression Models are used to examine dichotomous and count outcomes, respectively. All models control for state earned income tax credit rate, refundability of state earned income tax credit, Maximum food stamp allotment for a family of 3 maximum TANF allotment for a family of 3, 1-year lagged GDP, comprehensive Medicaid expansion, age, marital status, education and having minor children, year as well as state fixed-effects. All models are weighted for complex survey design and non-response. Total population models also control for gender. Standard errors are robust and clustered at the state level. Results of LPMs and PRMs are presented as percentage point differences in the probability of an outcome and Rate Ratios, respectively. All monetary values are inflation-adjusted. Boldface indicates statistical significance. Significance levels: *(access to care: *p*-value−.025, health behaviors: *p*-value−.0125, and health outcomes: *p*-value .010) (Bonferroni-corrected 95% confidence intervals in parenthesis). Notes: 1. Marginal Effect 2. Rate Ratio; Bold indicates statistical significance

## Discussion

This study examined the association between minimum wage policies, access to care, health behaviors and health outcomes, across race/ethnicity and gender by analyzing 21 years of data (1993–1914) from the BRFSS and other sources of state-level data. We found that in the total population, minimum wage increases were associated with improvements in access to care and dietary quality. In analyses conducted within race/ethnicity/gender subgroups we found that minimum wage increases were associated with improved access to care among white men, black women and Latino women but were associated with mixed impacts on the access to care of Latino men and negative impacts on access to care among white women and black men. With respect to dietary quality, minimum wage increases were associated with dietary improvements, mixed results and negative impacts among white, Latino and black men, respectively. Additionally, minimum wage increases were negatively linked to reports of no exercise and alcohol consumption among Latino and black men, respectively. With respect to health outcomes, minimum wage increases were negatively associated with reports of hypertension, poor physical health days and poor mental health days among white women but positively associated with poor mental health days among white men. Among Latino men, minimum wage increases were negatively associated with self-reported fair or poor health but positively associated with poor physical health days. This is one of the few studies, to our knowledge, to explore the health implications of minimum wage policies by both race/ethnicity and gender.

The findings regarding dietary quality are inconsistent with that of Horn et al.(2017) and Andreyeva et al.(2018) who find reduced dietary quality associated with minimum wage increases. The results likely diverge for a number of reasons. We use a different model specification for our count outcomes (PRM instead of OLS) as well as a different primary predictor (lagged real minimum wage vs. the adjusted wage ratio).

The findings of increased access to care among the total population are consistent with the findings of both McCarrier et al.(2011) and Andreyeva et al.(2018) who find reductions in the reporting of missed care due to cost among the total population and increases in well patient doctor visits among whites associated with minimum wage increases, respectively [12, 13]. These findings suggest that any associated drops in health benefits by employers to reduce labor cost, on average, were offset by either the purchase of health insurance in the individual market, expansions in access to public insurance, or both [30]. However, our analyses show that the access to care implications of minimum wage increases vary across race/ethnicity/gender subgroups.

This study finds reported health improvements associated with minimum wage increases among white women. Women constitute a larger proportion than men of workers either at or below minimum wage so women generally and white women particularly, have the potential for substantial financial benefit from minimum wage increases which may manifest in improved health outcomes [7].

Conversely, this study finds negative and mixed health outcomes reported among white and Latino men, respectively associated with minimum wage increases and a negative trend in health outcomes for black men. These findings are somewhat consistent with the work of Horn et al. (2017) that finds increases in self-reported fair/poor health among men associated with minimum wage increases. However, these findings are inconsistent with the work of Andreyeva et al.(2018) who do not find adverse health effects associated with minimum wage increases among minorities; however, since they combine blacks and Latinos and do not disaggregate the results by gender it is difficult to directly compare these results. Averett et al. (2017), the only study, to our knowledge, to examine the implications of minimum wage polices across both race/ethnicity (white, black, Latino) and gender, simultaneously, found that Latino male teenagers had increases in self-reported fair/poor health associated with minimum wage increases [14].

Economic dislocation, as a consequence of reduced labor demand, is one potential explanation for the negative association between reported health outcomes and minimum wage increases among men. Economic dislocation in the context of minimum wage increases may be particularly salient for individuals with the lowest levels of human capital [2]. Specifically, the most vulnerable workers with very low education levels, or limited English proficiency may be the first to lose employment if an increase in the minimum wage induces employers to cut jobs [31]. Additionally, racial/ethnic/gender subgroups that are the most highly concentrated in labor intensive sectors are particularly vulnerable to labor restructuring and job loss in the setting of increased labor costs. Relative to all other race/ethnicity/gender groups, Latino men are the most occupationally segregated in labor intensive sectors so they may face disproportionate negative economic impacts from minimum wage increases. By contrast, white men have the lowest level of occupational segregation and black men have an intermediate level of occupational segregation [8, 32]. Increases in access to care is another potential explanation for the worsened health outcomes reported by white and Latino men, as a consequence of increasing awareness of asymptomatic medical diseases [33].

### Limitations

The results of this study must be interpreted in the context of several limitations. The study is observational and could be subject to residual confounding. The shortcomings of the BRFSS with respect to potential selection bias have been previously documented; it is worth noting that underrepresentation of individuals without home telephones and those working atypical hours necessarily exclude individuals who may be disproportionately affected by the minimum wage [34]. Minimum wage changes undertaken by municipalities are not included. An important measurement limitation is the use of self-reported health measures, which have been shown to be a more reliable predictor of health outcomes in whites than among racial/ethnic minorities [33]. Additionally, due to changes in the way BLS categorized median wage categories after 1999 the primary predictor slightly changes after 1999. Given that this change is not correlated with minimum wage changes, it is unlikely that this change has significant implications for our results. BRFSS changed its sampling strategy with the 2011 wave, results are not formally comparable across years. Since the point of this analysis is not time trends but rather to use cross-state variation with year controls, this issue should not pose a problem for this analysis. These results—as is the case for almost all of the literature—examine only the short-term effects of minimum-wage increases. Longer term effects could differ substantially from those estimated here, but cannot be identified in empirical analyses without overly strong assumptions about their timing.

These limitations are balanced by some important contributions. Given that previous work on this topic has primarily focused on average population effects, it is useful to provide preliminary evidence that contributes to a more comprehensive understanding of how these policies operate among different portions of the low-wage work force.

## Conclusions

While increasing the minimum wage is associated with some improvement in access to care and dietary quality in certain subgroups and improved self-reported health outcomes among white women it may be linked to negative health outcomes among men. Although there appears to be enthusiasm for minimum-wage increases in the public health community, such increases may have to be paired with deliberate strategies to protect the workers that might be vulnerable to economic dislocation, for example with robust unemployment insurance and increased access to job training for displaced workers [35]. Future studies should look at the implications of minimum wage increases for health outcomes across racial/ethnic/gender groups using more valid measures of health status.

### Additional file

Additional file 1: Adjustment factors for all state-year combinations (1993-2014). (XLS 46 kb)

## Data Availability

The datasets analyzed during the current study are publicly available from the Centers for Disease Control and Prevention, https://www.cdc.gov/brfss/annual_data/annual_data.htm

## Appendix

**Table 7:** Adjusted Wage Ratio by Time & Census Region [with inter-quartile ranges]

	1993-1996	1997-2001	2002-2006	2007-2011	2012-2014
Northeast	4.19 [4.02 – 4.38]	4.90 [4.69 – 5.19]	5.45 [5.01 –6.04]	6.71 [6.30 – 7.24]	6.89 [6.39 – 7.30]
Midwest	4.43 [4.23 – 4.56]	5.29 [5.00 – 5.58]	5.38 [5.18 – 5.55]	6.92 [6.41 – 7.59]	7.72 [7.54 – 7.82]
South	4.54 [4.27 – 4.44]	5.57 [5.25 – 6.04]	5.70 [5.38 – 6.02]	6.85 [6.01 –7.56]	7.64 [7.16 – 7.95]
West	4.25 [3.87 – 4.53]	5.25 [4.92 – 5.69]	5.87 [5.49 – 6.22]	7.12 [6.63 –7.64]	7.81 [7.52 – 8.17]
All Regions, All Years	6.11 [3.18 - 9.37]				

The data source is BRFSS (1993-2014 panels). [Interquartile Range in brackets

**Table 8:** High vs. Low Wage in Minimum Wage, Access to Care, and Health (All Races)

Outcome	Total		Men		Women	
	High Wage	Low Wage	High Wage	Low Wage	High Wage	Low Wage
Access to Care						
No Health Insurance^1^	**-.02** *N*=747 642	-.00 *N*=121 789	**-.03** *N*=344 633	-.00 *N*=54 766	**-.02** *N*=403 009	-.00 *N*=67 023
Missed care due to cost^1^	**-.01** *N*=711 473	.01 *N*=110 914	**-.01** *N*=328 148	.01 *N*=49 719	**-.01** *N*=383 325	.02 *N*=61 195
Health Behavior						
No exercise^1^	-.00 *N*=664 499	.00 *N*=116 952	.00 *N*=305 791	.01 *N*=52 499	-.01 *N*=358 708	-.01 *N*=64 453
Fruit Consumption^2^	**1.03** *N*=389 051	1.13 *N*=56 648	1.04 *N*=177 883	1.29 *N*=25 077	1.02 *N*=211 168	.96 *N*=31 571
Vegetable Consumption^2^	**1.06** *N*=386 748	**.89** *N*=56 385	**1.06** *N*=176 504	**.89** *N*=24 959	**1.06** *N*=210 244	**.88** *N*=31 426
Alcohol Consumption^2^	1.01 *N*=476 376	1.03 *N*=78 405	1.01 *N*=235 502	1.04 *N*=38 315	1.02 *N*=240 874	.99 *N*=40 090
Health Outcomes						
Self-reported poor health^1^	.00 *N*=747 245	.01 *N*=121 735	.01 *N*=344 651	.01 *N*=54 747	-.00 *N*=402 594	.01 *N*=66 988
Self-reported HTN^1^	.00 *N*=410 673	.02 *N*=63 735	.01 *N*=188 893	.02 *N*=28 356	.00 *N*=221 780	.02 *N*=35 379
Unhealthy Days^2^	1.02 *N*=736 989	.99 *N*=120 881	1.04 *N*=340 186	.95 *N*=54 389	1.01 *N*=396 803	1.02 *N*=66 492
Poor Mental Health Days^2^	1.03 *N*=728 880	.96 *N*=119 555	1.06 *N*=336 682	.93 *N*=53 821	1.01 *N*=392 198	.98 *N*=65 734
Poor Physical Health Days^2^	1.02 *N*=729 333	1.05 *N*=119 552	1.03 *N*=336 990	1.00 *N*=53 839	1.01 *N*=392 343	1.11 *N*=65 713

The data source is BRFSS (1993-2014 panels). Linear Probability Models and Poisson Regression Models are used to examine dichotomous and count outcomes, respectively. All models control for state earned income tax credit rate, refundability of state earned income tax credit, Maximum food stamp allotment for a family of 3 maximum TANF allotment for a family of 3, 1-year lagged GDP, comprehensive Medicaid expansion, age, race/ethnicity, marital status, education and having minor children, year as well as state fixed-effects. All models are weighted for complex survey design and non-response. Total population models also control for gender. Standard errors are robust and clustered at the state level. Results of LPMs and PRMs are presented as percentage point differences in the probability of an outcome and Rate Ratios, respectively. All monetary values are inflation-adjusted. Boldface indicates statistical significance. Significance levels: *(access to care: *p*-value-.025, health behaviors: *p*-value-.0125, and health outcomes: *p*-value .010). Notes: 1. Marginal effect 2. Rate Ratio

**Table 9:** High vs. Low Wage in Minimum Wage, Access to Care, and Health (Whites)

Outcome	Total		Men		Women	
	High Wage	Low Wage	High Wage	Low Wage	High Wage	Low Wage
Access to Care						
No Health Insurance^1^	-.00 *N*=532 351	.00 *N*=91 312	-.00 *N*=247 036	-.00 *N*=42 089	-.01 *N*=285 315	.00 *N*=49 223
Missed care due to cost^1^	**-.01** *N*=507 490	.02 *N*=82 224	-.01 *N*=235 621	.01 *N*=37 785	**-.01** *N*=271 869	.03 *N*=44 439
Health Behavior						
No exercise^1^	.00 *N*=469 800	.01 *N*=87 238	**.01** *N*=217 393	.01 *N*=40 147	-.01 *N*=252 407	.01 *N*=47 091
Fruit Consumption^2^	1.02 *N*=279 360	1.23 *N*=42 729	1.03 *N*=128 598	1.48 *N*=19 366	1.01 *N*=150 762	1.00 *N*=23 363
Vegetable Consumption^2^	**1.04** *N*=278 403	**.87** *N*=42 620	**1.04** *N*=128 004	**.87** *N*=19 321	**1.04** *N*=150 399	**.86** *N*=23 299
Alcohol Consumption^2^	1.01 *N*=344 710	1.06 *N*=59 306	1.01 *N*=169 790	1.10 *N*=29 417	1.02 *N*=174 920	.95 *N*=29 889
Health Outcomes						
Self-reported poor health^1^	.00 *N*=532 128	.01 *N*=91 285	**.01** *N*=247 043	.00 *N*=42 071	**-.01** *N*=285 085	.01 *N*=49 214
Self-reported HTN^1^	-.00 *N*=296 142	.02 *N*=47 407	-.00 *N*=137 269	.03 *N*=21 677	-.00 *N*=158 873	.00 *N*=25 730
Unhealthy Days^2^	1.00 *N*=525 105	.99 *N*=90 634	1.02 *N*=243 959	.95 *N*=41 816	.99 *N*=281 146	1.05 *N*=48 818
Poor Mental Health Days^2^	.99 *N*=519 615	.98 *N*=89 654	1.01 *N*=241 610	.96 *N*=41 386	.98 *N*=278 005	1.00 *N*=48 268
Poor Physical Health Days^2^	1.02 *N*=520 554	1.07 *N*=89 774	1.05 *N*=242 059	.98 *N*=41 442	1.00 *N*=278 495	1.18 *N*=48 332

The data source is BRFSS (1993-2014 panels). Linear Probability Models and Poisson Regression Models are used to examine dichotomous and count outcomes, respectively. All models control for state earned income tax credit rate, refundability of state earned income tax credit, Maximum food stamp allotment for a family of 3 maximum TANF allotment for a family of 3, 1-year lagged GDP, comprehensive Medicaid expansion, age, marital status, education and having minor children, year as well as state fixed-effects. All models are weighted for complex survey design and non-response. Total population models also control for gender. Standard errors are robust and clustered at the state level. Results of LPMs and PRMs are presented as percentage point differences in the probability of an outcome and Rate Ratios, respectively. All monetary values are inflation-adjusted. Boldface indicates statistical significance. Significance levels: *(access to care: *p*-value-.025, health behaviors: *p*-value-.0125, and health outcomes: *p*-value .010). Notes: 1. Marginal effect 2. Rate Ratio

**Table 10:** High vs. Low Wage in Minimum Wage, Access to Care, and Health (Blacks)

Outcome	Total		Men		Women	
	High Wage	Low Wage	High Wage	Low Wage	High Wage	Low Wage
Access to Care						
No Health Insurance^1^	.00 *N*=80 063	.07 *N*=14 841	.02 *N*=29 619	.09 *N*=5 059	-.01 *N*=50 444	.03 *N*=9 782
Missed care due to cost^1^	.01 *N*=75 887	.02 *N*=14 021	.01 *N*=28 083	.05 *N*=4 760	.00 *N*=47 804	-.01 *N*=9 261
Health Behavior						
No exercise^1^	-.01 *N*=70 615	-.05 *N*=14 440	-.01 *N*=26 053	-.03 *N*=4 901	-.00 *N*=44 562	-.06 *N*=9 539
Fruit Consumption^2^	1.01 *N*=41 414	.97 *N*=6 580	.92 *N*=15 280	.86 *N*=2 177	1.08 *N*=26 134	.99 *N*=4 403
Vegetable Consumption^2^	1.01 *N*=41 315	1.28 *N*=6 523	1.00 *N*=15 242	1.49 *N*=2 151	1.03 *N*=26 073	1.12 *N*=4 372
Alcohol Consumption^2^	.97 *N*=46 273	1.26 *N*=8 751	.94 *N*=18 750	1.17 *N*=3 388	1.05 *N*=27 523	1.29 *N*=5 363
Health Outcomes						
Self-reported poor health^1^	.02 *N*=79 965	.02 *N*=14 828	.02 *N*=29 606	.05 *N*=5 061	.01 *N*=50 359	-.02 *N*=9 767
Self-reported HTN^1^	-.00 *N*=44 111	.01 *N*=7 982	.03 *N*=16 397	-.05 *N*=2 677	-.03 *N*=27 714	.07 *N*=5 305
Unhealthy Days^2^	1.00 *N*=78 195	1.16 *N*=14 716	1.02 *N*=28 964	1.33 *N*=5 014	.98 *N*=49 231	1.03 *N*=9 702
Poor Mental Health Days^2^	1.03 *N*=77 364	1.17 *N*=14 563	1.11 *N*=28 639	1.38 *N*=4 962	.97 *N*=48 725	1.06 *N*=9 601
Poor Physical Health Days^2^	1.00 *N*=77 026	1.11 *N*=14 474	.99 *N*=28 568	1.14 *N*=4 935	1.01 *N*=48 458	1.04 *N*=9 539

The data source is BRFSS (1993-2014 panels). Linear Probability Models and Poisson Regression Models are used to examine dichotomous and count outcomes, respectively. All models control for state earned income tax credit rate, refundability of state earned income tax credit, Maximum food stamp allotment for a family of 3 maximum TANF allotment for a family of 3, 1-year lagged GDP, comprehensive Medicaid expansion, age, marital status, education and having minor children, year as well as state fixed-effects. All models are weighted for complex survey design and non-response. Total population models also control for gender. Standard errors are robust and clustered at the state level. Results of LPMs and PRMs are presented as percentage point differences in the probability of an outcome and Rate Ratios, respectively. All monetary values are inflation-adjusted. Boldface indicates statistical significance. Significance levels: *(access to care: *p*-value-.025, health behaviors: *p*-value-.0125, and health outcomes: *p*-value .010). Notes: 1. Marginal effect 2. Rate Ratio

**Table 11:** High vs. Low Wage in Minimum Wage, Access to Care, and Health (Latinos)

Outcome	Total		Men		Women	
	High Wage	Low Wage	High Wage	Low Wage	High Wage	Low Wage
Access to Care						
No Health Insurance^1^	-.02 *N*=72 897	-.07 *N*=8 389	-.03 *N*=36 663	-.12 *N*=4 146	-.00 *N*=36 234	-.01 *N*=4 243
Missed care due to cost^1^	-.00 *N*=68 767	.02 *N*=7 962	-.01 *N*=34 588	.08 *N*=3 958	.02 *N*=34 179	-.07 *N*=4 004
Health Behavior						
No exercise^1^	.00 *N*=71 610	.03 *N*=8 245	.00 *N*=35 970	.13 *N*=4 079	-.00 *N*=35 640	-.11 *N*=4 166
Fruit Consumption^2^	**1.10** *N*=34 305	.69 *N*=3 730	**1.15** *N*=17 066	.57 *N*=1 822	1.03 *N*=17 239	.96 *N*=1 908
Vegetable Consumption^2^	1.05 *N*=33 519	**.61** *N*=3 667	1.05 *N*=16 597	**.51** *N*=1 790	1.04 *N*=16 922	.84 *N*=1 877
Alcohol Consumption^2^	.99 *N*=50 567	.68 *N*=5 831	.99 *N*=27 824	.65 *N*=3 135	.94 *N*=22 743	1.07 *N*=2 696
Health Outcomes						
Self-reported poor health^1^	-.00 *N*=72 733	.04 *N*=8 380	-.01 *N*=36 596	.06 *N*=4 138	.01 *N*=36 137	.01 *N*=4 242
Self-reported HTN^1^	.00 *N*=38 223	-.08 *N*=4 353	-.01 *N*=19 112	-.10 *N*=2 113	.03 *N*=19 111	-.05 *N*=2 240
Unhealthy Days^2^	**1.12** *N*=71 783	.88 *N*=8 346	1.15 *N*=36 092	1.03 *N*=4 120	1.09 *N*=35 691	.71 *N*=4 226
Poor Mental Health Days^2^	1.11 *N*=70 869	.69 *N*=8 258	1.14 *N*=35 666	.80 *N*=4 076	1.07 *N*=35 203	.57 *N*=4 182
Poor Physical Health Days^2^	**1.17** *N*=70 728	.97 *N*=8 235	**1.20** *N*=35 586	1.32 *N*=4 073	1.13 *N*=35 142	.66 *N*=4 162

The data source is BRFSS (1993-2014 panels). Linear Probability Models and Poisson Regression Models are used to examine dichotomous and count outcomes, respectively. All models control for state earned income tax credit rate, refundability of state earned income tax credit, Maximum food stamp allotment for a family of 3 maximum TANF allotment for a family of 3, 1-year lagged GDP, comprehensive Medicaid expansion, age, marital status, education and having minor children, year as well as state fixed-effects. All models are weighted for complex survey design and non-response. Total population models also control for gender. Standard errors are robust and clustered at the state level. Results of LPMs and PRMs are presented as percentage point differences in the probability of an outcome and Rate Ratios, respectively. All monetary values are inflation-adjusted. Boldface indicates statistical significance. Significance levels: *(access to care: *p*-value-.025, health behaviors: *p*-value-.0125, and health outcomes: *p*-value .010). Notes: 1. Marginal effect 2. Rate Ratio

**Table 12:** Marital Status and Relationship to Minimum Wage, Access to Care, and Health (All Races)

Outcome	Total		Men		Women	
	Married	Single	Married	Single	Married	Single
Access to Care						
No Health Insurance^1^	**-.02** *N*=486 874	**-.02** *N*=383 034	-.03 *N*=235 988	**-.03** *N*=163 616	-.02 *N*=250 886	-.01 *N*=219 418
Missed care due to cost^1^	-.00 *N*=460 731	**-.02** *N*=362 133	-.00 *N*=223 198	**-.02** *N*=154 873	-.00 *N*=237 533	-.01 *N*=207 260
Health Behavior						
No exercise^1^	-.00 *N*=436 872	-.00 *N*=345 057	.01 *N*=211 280	-.00 *N*=147 215	-.01 *N*=225 592	-.00 *N*=197 842
Fruit Consumption^2^	1.03 *N*=251 012	1.03 *N*=195 150	1.05 *N*=120 685	1.02 *N*=82 472	1.01 *N*=130 327	1.04 *N*=112 678
Vegetable Consumption^2^	**1.04** *N*=250 015	**1.06** *N*=193 587	**1.05** *N*=120 005	1.06 *N*=81 658	**1.04** *N*=130 010	**1.07** *N*=111 929
Alcohol Consumption^2^	1.02 *N*=306 208	.99 *N*=248 573	1.02 *N*=158 076	.98 *N*=115 741	1.00 *N*=148 132	1.03 *N*=132 832
Health Outcomes						
Self-reported poor health^1^	-.00 *N*=486 541	.01 *N*=382 916	.00 *N*=235 872	.01 *N*=163 731	-.00 *N*=250 669	-.00 *N*=219 185
Self-reported HTN^1^	.00 *N*=266 322	.01 *N*=208 086	.00 *N*=128 816	.01 *N*=88 433	.00 *N*=137 506	.00 *N*=119 653
Unhealthy Days^2^	1.03 *N*=480 726	1.01 *N*=377 619	1.03 *N*=233 165	1.02 *N*=161 614	1.02 *N*=247 561	1.00 *N*=216 005
Poor Mental Health Days^2^	1.05 *N*=476 342	1.01 *N*=372 555	1.07 *N*=231 271	1.02 *N*=159 433	1.02 *N*=245 071	.99 *N*=213 122
Poor Physical Health Days^2^	1.01 *N*=476 413	1.02 N=372 941	1.01 *N*=231 279	1.04 *N*=159 752	1.02 *N*=245 134	1.01 *N*=213 189

The data source is BRFSS (1993-2014 panels). Linear Probability Models and Poisson Regression Models are used to examine dichotomous and count outcomes, respectively. All models control for state earned income tax credit rate, refundability of state earned income tax credit, Maximum food stamp allotment for a family of 3 maximum TANF allotment for a family of 3, 1-year lagged GDP, comprehensive Medicaid expansion, age, race/ethnicity, education and having minor children, year as well as state fixed-effects. All models are weighted for complex survey design and non-response. Total population models also control for gender. Standard errors are robust and clustered at the state level. Results of LPMs and PRMs are presented as percentage point differences in the probability of an outcome and Rate Ratios, respectively. All monetary values are inflation-adjusted. Boldface indicates statistical significance. Significance levels: *(access to care: *p*-value-.025, health behaviors: *p*-value-.0125, and health outcomes: *p*-value .010) Notes: 1. Marginal effect 2. Rate Ratio; Bold indicates statistical significance.

**Table 13:** Marital Status and Relationship to Minimum Wage, Access to Care, and Health (Whites)

Outcome	Total		Men		Women	
	Married (mr)	Single (sg)	Married (mr)	Single (sg)	Married (mr)	Single (sg)
Access to Care						
No Health Insurance^1^	-.00 *N*=376 398	-.00 *N*=247 534	-.00 *N*=178 021	-.01 *N*=11 233	-.01 *N*=198 377	-.00 *N*=136 301
Missed care due to cost^1^	-.01 *N*=356 354	-.01 *N*=233 629	-.00 *N*=168 404	-.01 *N*=105 131	-.01 *N*=187 950	-.01 *N*=128 498
Health Behavior						
No exercise^1^	.01 *N*=335 959	.00 *N*=221 348	.02 *N*=158 379	.01 *N*=99 290	-.01 *N*=177 580	-.01 *N*=122 058
Fruit Consumption^2^	1.02 *N*=195 182	1.03 *N*=127 169	1.03 *N*=91 720	1.05 *N*=56 368	1.01 *N*=103 462	1.01 *N*=70 801
Vegetable Consumption^2^	1.02 *N*=194 897	**1.05** *N*=126 391	1.03 *N*=91 526	1.05 *N*=55 926	1.02 *N*=103 371	**1.05** *N*=70 465
Alcohol Consumption^2^	1.00 *N*=239 901	1.01 *N*=164 115	1.01 *N*=120 002	1.01 *N*=79 205	.99 *N*=119 899	1.04 *N*=84 910
Health Outcomes						
Self-reported poor health^1^	-.00 *N*=376 165	.00 *N*=247 517	.01 *N*=177 921	.01 *N*=111 322	-.01 *N*=198 244	-.01 *N*=136 195
Self-reported HTN^1^	.00 *N*=207 719	.00 *N*=135 830	.00 *N*=98 205	.00 *N*=60 741	.00 *N*=109 514	.00 *N*=75 089
Unhealthy Days^2^	1.02 *N*=371 785	.99 *N*=244 223	1.03 *N*=175 962	1.00 *N*=109 942	1.00 *N*=195 823	.98 *N*=134 281
Poor Mental Health Days^2^	1.01 *N*=368 657	.98 *N*=240 874	1.02 *N*=174 689	.98 *N*=108 435	1.01 *N*=193 968	.97 *N*=132 439
Poor Physical Health Days^2^	1.02 *N*=368 973	1.03 *N*=241 622	1.04 *N*=174 726	1.04 *N*=108 833	.99 *N*=194 177	1.02 *N*=132 789

The data source is BRFSS (1993-2014 panels). Linear Probability Models and Poisson Regression Models are used to examine dichotomous and count outcomes, respectively. All models control for state earned income tax credit rate, refundability of state earned income tax credit, Maximum food stamp allotment for a family of 3 maximum TANF allotment for a family of 3, 1-year lagged GDP, comprehensive Medicaid expansion, age, education and having minor children, year as well as state fixed-effects. All models are weighted for complex survey design and non-response. Total population models also control for gender. Standard errors are robust and clustered at the state level. Results of LPMs and PRMs are presented as percentage point differences in the probability of an outcome and Rate Ratios, respectively. All monetary values are inflation-adjusted. Boldface indicates statistical significance. Significance levels: *(access to care: *p*-value-.025, health behaviors: *p*-value-.0125, and health outcomes: *p*-value .010) Notes: 1. Marginal effect 2. Rate Ratio

**Table 14:** Marital Status and Relationship to Minimum Wage, Access to Care, and Health (Blacks)

Outcome	Total		Men		Women	
	Married (mr)	Single (sg)	Married (mr)	Single (sg)	Married (mr)	Single (sg)
Access to Care						
No Health Insurance^1^	.00 *N*=31 203	.01 *N*=63 888	.01 *N*=14 829	.03 *N*=19 917	-.01 *N*=16 374	-.01 *N*=43 971
Missed care due to cost^1^	.01 *N*=29 568	.00 *N*=60 528	.01 *N*=14 053	.00 *N*=18 858	.01 *N*=15 515	-.00 *N*=41 670
Health Behavior						
No exercise^1^	.02 *N*=27 859	-.02 *N*=57 384	.03 *N*=13 154	-.03 *N*=17 868	.00 *N*=14 705	-.00 *N*=39 516
Fruit Consumption^2^	1.02 *N*=15 928	.99 *N*=32 246	.98 *N*=7 529	.86 *N*=9 993	1.05 *N*=8 399	1.09 *N*=22 253
Vegetable Consumption^2^	1.02 *N*=15 895	1.01 *N*=32 127	1.06 *N*=7 514	.98 *N*=9 945	.98 *N*=8 381	1.04 *N*=22 182
Alcohol Consumption^2^	.97 *N*=17 247	.98 *N*=37 777	.96 *N*=8 918	.94 *N*=13 220	1.00 *N*=8 329	1.07 *N*=24 557
Health Outcomes						
Self-reported poor health^1^	.02 *N*=31 183	.01 *N*=63 797	.04 *N*=14 816	.00 N=19 919	-.00 *N*=16 367	.02 *N*=43 878
Self-reported HTN^1^	.02 *N*=17 124	-.01 *N*=34 969	.06 *N*=8 181	.02 *N*=10 893	-.03 *N*=8 943	-.03 *N*=24 076
Unhealthy Days^2^	1.03 *N*=30 549	1.00 *N*=62 548	1.03 *N*=14 532	1.01 *N*=19 514	1.01 *N*=16 017	1.00 *N*=43 034
Poor Mental Health Days^2^	1.11 *N*=30 238	1.01 *N*=61 870	1.18 *N*=14 382	1.07 *N*=19 285	1.02 *N*=15 856	.97 *N*=42 585
Poor Physical Health Days^2^	1.00 *N*=30 095	1.01 *N*=61 587	.95 *N*=14 324	.99 *N*=19 246	1.06 *N*=15 771	1.03 *N*=42 341

The data source is BRFSS (1993-2014 panels). Linear Probability Models and Poisson Regression Models are used to examine dichotomous and count outcomes, respectively. All models control for state earned income tax credit rate, refundability of state earned income tax credit, Maximum food stamp allotment for a family of 3 maximum TANF allotment for a family of 3, 1-year lagged GDP, comprehensive Medicaid expansion, age, education and having minor children, year as well as state fixed-effects. All models are weighted for complex survey design and non-response. Total population models also control for gender. Standard errors are robust and clustered at the state level. Results of LPMs and PRMs are presented as percentage point differences in the probability of an outcome and Rate Ratios, respectively. All monetary values are inflation-adjusted. Boldface indicates statistical significance. Significance levels: *(access to care: *p*-value-.025, health behaviors: *p*-value-.0125, and health outcomes: *p*-value .010) Notes: 1. Marginal effect 2. Rate Ratio

**Table 15:** Marital status and relationship to minimum wage, access to care, and health (Latinos)

Outcome	Total		Men		Women	
	Married (mr)	Single (sg)	Married (mr)	Single (sg)	Married (mr)	Single (sg)
Access to Care						
No Health Insurance^1^	-.02 *N*=43 470	-.02 *N*=37 816	-.01 *N*=24 170	**-.05** *N*=16 639	-.03 *N*=19 300	.01 *N*=21 177
Missed care due to cost^1^	.03 *N*=40 941	**-.03** *N*=35 788	.02 *N*=22 751	**-.05** *N*=15 795	**.05** *N*=18 190	-.00 *N*=19 993
Health Behavior						
No exercise^1^	-.01 *N*=42 775	.01 *N*=37 080	-.00 *N*=23 772	.01 *N*=16 277	-.02 *N*=19 003	.02 *N*=20 803
Fruit Consumption^2^	1.09 *N*=20 366	1.14 *N*=17 669	1.18 *N*=11 181	1.15 *N*=7 707	.94 *N*=9 185	1.14 *N*=9 962
Vegetable Consumption^2^	1.01 *N*=19 932	1.08 *N*=17 254	1.01 *N*=10 880	1.09 *N*=7 507	1.00 *N*=9 052	1.07 *N*=9 747
Alcohol Consumption^2^	.98 *N*=29 723	.96 *N*=26 675	.99 *N*=17 970	.97 *N*=12 989	.89 *N*=11 753	.94 *N*=13 686
Health Outcomes						
Self-reported poor health^1^	-.01 *N*=43 371	.01 *N*=37 742	-.02 *N*=24 133	.01 *N*=16 601	.01 *N*=19 238	.01 *N*=21 141
Self-reported HTN^1^	-.02 *N*=22 818	.02 *N*=19 758	-.03 *N*=12 580	.01 *N*=8 645	.01 *N*=10 238	.04 *N*=11 113
Unhealthy Days^2^	1.15 *N*=42 866	1.07 *N*=37 263	1.17 *N*=23 823	1.09 *N*=16 389	1.11 *N*=19 043	1.07 *N*=20 874
Poor Mental Health Days^2^	1.15 *N*=42 371	1.05 *N*=36 756	1.20 *N*=23 569	1.05 *N*=16 173	1.08 *N*=18 802	1.06 *N*=20 583
Poor Physical Health Days^2^	1.19 *N*=42 277	1.12 *N*=36 686	1.20 *N*=23 524	1.18 *N*=16 135	1.17 *N*=18 753	1.08 *N*=20551

The data source is BRFSS (1993-2014 panels). Linear Probability Models and Poisson Regression Models are used to examine dichotomous and count outcomes, respectively. All models control for state earned income tax credit rate, refundability of state earned income tax credit, Maximum food stamp allotment for a family of 3 maximum TANF allotment for a family of 3, 1-year lagged GDP, comprehensive Medicaid expansion, age, education and having minor children, year as well as state fixed-effects. All models are weighted for complex survey design and non-response. Total population models also control for gender. Standard errors are robust and clustered at the state level. Results of LPMs and PRMs are presented as percentage point differences in the probability of an outcome and Rate Ratios, respectively. All monetary values are inflation-adjusted. Boldface indicates statistical significance. Significance levels: *(access to care: *p*-value-.025, health behaviors: *p*-value-.0125, and health outcomes: *p*-value .010) Notes: 1. Marginal effect 2. Rate Ratio

## Abbreviations

BLS: Bureau of Labor Statistics
BRFSS: Behavioral Risk Factor Surveillance System
LPM: Linear Probability Model
PRM: Linear Regression Models and Poisson Regression Models
TANF: Temporary Assistance for Needy Families
